# “I have no disease and weed just relaxes me!”: The therapeutic challenge in young patients with psychosis and cannabis abuse

**DOI:** 10.1192/j.eurpsy.2021.456

**Published:** 2021-08-13

**Authors:** P. Mota

**Affiliations:** Departamento De Psiquiatria E Saúde Mental, Centro Hospitalar do Tâmega e Sousa, Guilhufe, Portugal

**Keywords:** Cannabis, Substance use disorders, psychosis

## Abstract

**Introduction:**

Substance use disorders (SUDs) are estimated to affect around 30 million people worldwide, and are characterized by repeated use of a substance that leads to clinically significant impairment or suffering, making it a serious health problem, with high associated costs.

**Objectives:**

Understand and evaluate the impact of cannabis use on adherence to treatment in young patients with psychosis.

**Methods:**

Narrative literature review by performing a search on MedLine for English-written articles. The query used was “(Cannabis) AND (Schizophrenia OR Psychosis) AND (Adherence)”.

**Results:**

About 70 to 80% of young people with SUDs have at least one concomitant psychiatric disorder and cannabis is involved in approximately 50% of psychosis or schizophrenia of those cases, so there is a growing concern about the deleterious medical and psychiatric consequences of the increase and early initiation of consumption of this substance. It is estimated that about 26% of patients with psychotic conditions do not adhere to the treatment plan established by the psychiatrist; however, especially during the inaugural phases of psychotic disorders, rates of non-adherence to therapy are high (above 50%), and are said to be higher in younger patients.

**Conclusions:**

The risk of relapse after a first psychotic episode is high. As the use of cannabis is a potentially preventable risk factor, interventions aimed at improving therapeutic adherence in psychotic conditions must specifically target the use of this substance, since reducing its consumption can lead to a more favorable course of the disease and at less expensive costs in addressing these pathologies.

**Disclosure:**

No significant relationships.

